# Polylactic Glycolic Acid-Mediated Delivery of Plectasin Derivative NZ2114 in *Staphylococcus epidermidis* Biofilms

**DOI:** 10.3390/antibiotics13030228

**Published:** 2024-02-29

**Authors:** Xuanxuan Ma, Na Yang, Ruoyu Mao, Ya Hao, Da Teng, Yinhua Huang, Jianhua Wang

**Affiliations:** 1 Innovative Team of Antimicrobial Peptides and Alternatives to Antibiotics, Gene Engineering Laboratory, Feed Research Institute, Chinese Academy of Agricultural Sciences, Beijing 100081, China; 82101209114@caas.cn (X.M.); yangna@caas.cn (N.Y.); haoya@caas.cn (Y.H.); tengda@caas.cn (D.T.); 2 State Key Laboratory of Farm Animal Biotech Breeding, College of Biology Sciences, China Agricultural University, Beijing 100193, China; 3 Key Laboratory of Feed Biotechnology, Ministry of Agriculture and Rural Affairs, Beijing 100081, China

**Keywords:** antimicrobial peptide NZ2114, drug delivery system, nanoparticles, PLGA, *Staphylococcus epidermidis*, biofilms

## Abstract

Antimicrobial peptides (AMPs) are antibiotic candidates; however, their instability and protease susceptibility limit clinical applications. In this study, the polylactic acid–glycolic acid (PLGA)–polyvinyl alcohol (PVA) drug delivery system was screened by orthogonal design using the double emulsion–solvent evaporation method. NZ2114 nanoparticles (NZ2114-NPs) displayed favorable physicochemical properties with a particle size of 178.11 ± 5.23 nm, polydispersity index (PDI) of 0.108 ± 0.10, ζ potential of 4.78 ± 0.67 mV, actual drug-loading rate of 4.07 ± 0.37%, encapsulation rate of 81.46 ± 7.42% and cumulative release rate of 67.75% (120 h) in PBS. The results showed that PLGA encapsulation increased HaCaT cell viability by 20%, peptide retention in 50% serum by 24.12%, and trypsin tolerance by 4.24-fold. Meanwhile, in vitro antimicrobial assays showed that NZ2114-NPs had high inhibitory activity against *Staphylococcus epidermidis* (*S. epidermidis*) (4–8 μg/mL). Colony counting and confocal laser scanning microscopy (CLSM) confirmed that NZ2114-NPs were effective in reducing the biofilm thickness and bacterial population of *S. epidermidis* G4 with a 99% bactericidal rate of persister bacteria, which was significantly better than that of free NZ2114. In conclusion, the results demonstrated that PLGA nanoparticles can be used as a reliable NZ2114 delivery system for the treatment of biofilm infections caused by *S. epidermidis*.

## 1. Introduction

The misuse of antibiotics has greatly exacerbated the development of bacterial resistance, and the formation of bacterial biofilms, which allow bacteria to evade the effects of antibiotics on the bacteria within the biofilm, has further exacerbated the resistance situation, posing a major threat to public health safety [1,2,3]. It has been reported that the antibiotic resistance will directly lead to 1.27 million deaths in 2019, and more unfortunately, this number will increase to 10 million if more effective options do not emerge, resulting in USD 100 trillion in economic losses [4]. Although this estimate is still being debated, there is no doubt that the development of novel antimicrobial drugs to combat the antibiotic resistance crisis, especially as published by the World Health Organization for multi-resistant bacterial infections, is a top priority [5,6].

Antimicrobial peptides (AMPs), as a promising new class of antimicrobial drugs, widely exist in various organisms, and they constitute the first line of defense of the organism against exogenous infection [7,8]. More importantly, unlike traditional antibiotics that target receptor genes/ligands to kill bacteria, most AMPs rely on their hydrophobicity and cationic properties to act on bacterial cell membranes to kill bacteria, which means that microorganisms need to redesign their membrane structure to resist AMPs, which is undoubtedly costly. Therefore, it is more difficult to induce bacterial resistance to AMPs than to traditional antibiotics, making AMPs a hotspot for antimicrobial drug development [7,8,9,10]. However, the cytotoxicity and protease susceptibility of AMPs have limited their clinical application [11]. In detail, they have been facing challenges as pharmacokinetics, T_1/2_, and C_max_ cannot be well assayed [12,13]. In particular, the failure of pexiganan and iseganan in phase III clinical trials has raised concerns about the prospects of AMP conversion [14]. NZ2114 (Table 1), a derivative of Plectasin, showed an excellent antibacterial activity against Gram-positive bacteria, especially *Staphylococcus aureus* (*S. aureus*) and *Staphylococcus epidermidis* (*S. epidermidis*), with the minimum inhibitory concentration (MIC) as low as 4 μg/mL. However, it is not resistant to trypsin and has a low druggability in mature biofilm [15,16]. Therefore, it is urgent to design a reasonable drug delivery system (DDS) to improve the stability of AMP and the accessibility of delivery in biofilm in order to improve the bioavailability of AMP [17,18].

Among various drug delivery systems, polylactic acid–glycolic acid (PLGA), as a biodegradable aliphatic polyester copolymer [19], has been approved by the U.S. Food and Drug Administration (FDA) and the European Medicines Agency (EMA). At the same time, its excellent biocompatibility and sound mechanical strength have made it a highly sought-after macromolecule in the field of drug delivery (especially in proteins and peptides) [20,21]. Numerous studies have shown that PLGA as an AMP delivery system has the following advantages: (i) it can achieve long-lasting release of AMPs at the target site, (ii) it can alleviate the degradation by hydrolytic enzymes and proteases, and (iii) it reduces cytotoxicity in mammals [22,23]. For example, PLGA-encapsulated colistin can eradicate *Pseudomonas aeruginosa* biofilm [24]. The PLGA-constructed ESC nanodelivery system achieved controlled re-release in the lung environment and improved the therapeutic efficacy of ESC for the treatment of lung infections caused by *Pseudomonas aeruginosa* [25]. The PLGA delivery system of SAAP-148 achieved a prolonged release of SAAP-148 in biofilm, improved the selectivity index, and effectively reduced the cytotoxicity of SAAP-148 [26], so it is reasonable to speculate that the use of PLGA as a delivery system for NZ2114 could improve stability and delivery downstream efficiency in biofilms. However, the ratio of polylactic acid to polyglycolic acid (L/G) as well as the molecular weight affect the specific properties and degradation rate of the PLGA matrix and thus the release rate of the PLGA drug product [21], and so far, the properties of the optimal PLGA for AMP encapsulation have been less studied.

In this study, different types of PLGA as carrier materials and surfactant PVA as stabilizers were used to prepare NZ2114 nanoparticles with different drug-loading rates. The formulation was optimized by an orthogonal test, its physical and chemical properties and release rate were investigated to screen out the optimum formulation, and its safety, stability, and antimicrobial activity were systematically studied.

## 2. Results

### 2.1. NZ2114-NP Characterization

#### 2.1.1. Size and Zeta Potential of NZ2114-NP

PLGA nanoparticles loaded with NZ2114 were developed by the double emulsion solvent evaporation method [23]. Based on the fact that ratio of L:G in polylactic acid–glycolic acid (PLGA), polyvinyl alcohol (PVA) viscosity, and drug concentration play important roles in regulating particle formation size, drug loading, and drug release kinetics, orthogonal experiments were carried out with this condition to screen the optimal NZ2114-NP formulation. The results are shown in Table 1: the size distribution of nanoparticles (theoretical drug loading: 0.625%, formulations 1–4) was 165.05–378.50 nm, with PDI ranging from 0.045 to 0.225 and the ζ potential ranging from −22.3 to −12.6 mV; the size distribution of nanoparticles (theoretical drug loading: 1.25%, formulations 5–8) was 186.69–365.45 nm, the PDI range was 0.083–0.325, the ζ potential range was −12.13 to −9.56 mV; the size distribution of nanoparticles (theoretical drug loading: 2.5%, formulations 9–12) was 169.67–487.67 nm, the PDI range was 0.125–0.240, the ζ potential range was −5.93–2.56 mV; the size distribution of nanoparticles (theoretical drug loading: 5% formulations 13–16) was 178.11–412.40 nm, the PDI range was 0.108–0.250, and the ζ potential range was 4.78–8.24 mV; the results showed that the ζ potential increased with the increase in drug loading, which is in agreement with the previous studies [23].

#### 2.1.2. NZ2114-NP Drug-Loading and Encapsulation Rate

As shown in Table 2, the actual loading rate of nanoparticle formulations 1–4 was 0.57–0.60%, and the encapsulation rate was 91.5–96.18%; the actual loading rate of nanoparticle formulations 5–8 was 1.12–1.18%, and the encapsulation rate was 89.91–94.48%; the actual drug loading of nanoparticle formulations 9–12 was 2.01–2.19%, and the encapsulation rate was 91.5–96.18%; the actual drug loading of nanoparticle formulations 13–16 was 3.79–4.08%, and the encapsulation rate was 75.87–81.63%. The results showed that encapsulation efficiency decreased as the theoretical drug loading increased.

#### 2.1.3. NZ2114-NP Release Rate

**Figure 1:**
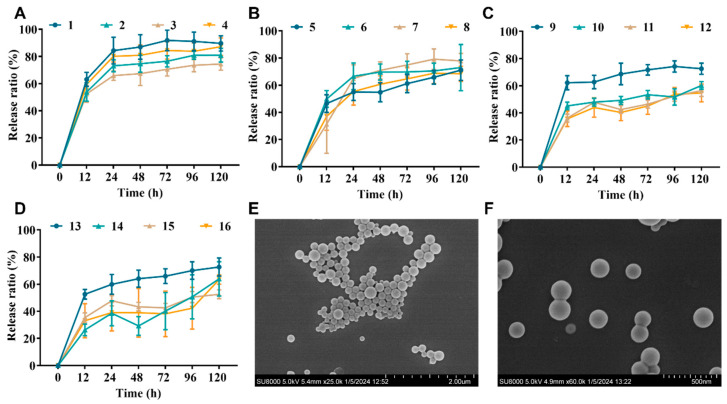
NP properties of PLGA-encapsulated NZ2114. (**A**–**D**) Cumulative release rates of NZ2114 from PLGA nanoparticles in phosphate buffer pH 7.4 ((**A**) loading: 0.625%; (**B**) loading: 1.25%; (**C**) loading: 2.5%; (**D**) loading: 5.0%) (mean ± SD, *n* = 4). (**E**,**F**) NPs (13) in different SEM plots with a magnification of 25 k and 60 k, respectively.

The initial release of most PLGA formulations is high, typically consuming one-quarter of the total drug on the first day. As shown in Figure 1, the cumulative release rates of nanoparticles with a theoretical drug loading of 0.625% (formulations 1–4) at 24 h and 120 h ranged 69.79–88.20% and 78.33–93.55%; those of nanoparticle formulations 5–8 (loading: 1.25%) at 24 h and 120 h ranged 54.91–66.60% and 68.37–77.94%; the cumulative release of nanoparticle formulations 9–12 at 24 h and 120 h was 43.50–58.43% and 52.11–68.27%; and the cumulative release of nanoparticle formulations 13–16 at 24 h and 120 h was 33.69–54.95% and 47.62–67.75%. The release profiles of all formulations showed a biphasic pattern with an initial burst release phase and a sustained release phase, which is in agreement with the results of the previous study [27].

Combining the size distribution, drug loading, and drug release, the optimal formulation was 13, which showed a theoretical drug loading of 5% and an actual loading of 4.07%, a ζ potential of 4.78 mV, a particle size of 178.11 nm, and a PDI of 0.108. The size distribution and uniformity were further verified by SEM (Figure 1E,F). The encapsulation efficiency was 81.46%, and the drug release rate was 54.95–67.75% at 24 h and 120 h, respectively. Formulation 13 will be used as the NZ2114-NP formulation in the subsequent studies.

### 2.2. Hemolysis Analysis

**Figure 2:**
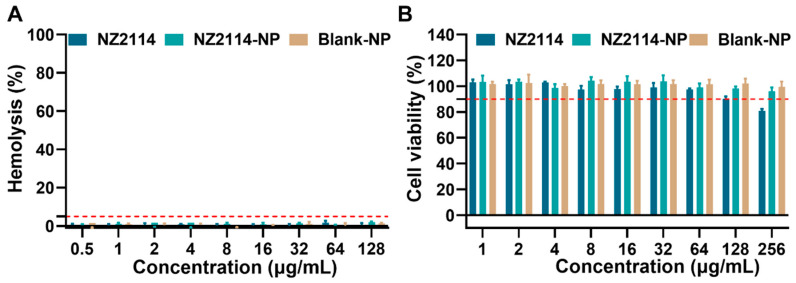
Safety of NZ2114 and NZ2114-NPs. (**A**) Hemolytic effect of NZ2114 and NZ2114-NPs against mouse erythrocytes; (**B**) Cytotoxicity of NZ2114 and NZ2114-NPs against HaCaT cells. NZ2114-NPs: PLGA-encapsulated NZ2114 (formulation-13), Blank NPs: formulation-13 without NZ2114. (**A**) Red line: 5% hemolysis rate; (**B**) red line: 90% cell viability. Results are mean ± SD (*n* = 3).

Subsequently, we assessed the hemolysis of NZ2114-NPs compared to the free peptide NZ2114. The results are shown in Figure 2A: the hemolysis rates of NZ2114 and NZ2114-NPs (128 μg/mL) were 0.67% and 1.87%, respectively, which were less than 5%, which was consistent with previous study [28]. Meanwhile, the hemolysis rate of the blank NPs was 1.27%, indicating that PLGA encapsulation did not increase the hemolytic load of NZ2114, and PLGA can be safely used as a packaging material.

### 2.3. Cytotoxicity

We further evaluated the cytotoxicity of NZ2114 NPs and free peptide NZ2114 on HaCaT cells. The results showed that the survival rate of HaCaT cells in the presence of NZ2114 (256 μg/mL, 12 h) was 80.78%, indicating that high concentrations of NZ2114 were potentially toxic to HaCaT cells. It is worth noting that the survival rate of HaCaT cells in the presence of NZ2114-NPs (256 μg/mL) was 96.03%. The PLGA encapsulation of NZ2114 reduced the cytotoxicity by 20% against HaCaT cells, and the Blank NPs showed no cytotoxicity (Figure 2B). These results showed that PLGA encapsulation significantly reduced the cytotoxicity of NZ2114.

### 2.4. Serum Stability

**Figure 3:**
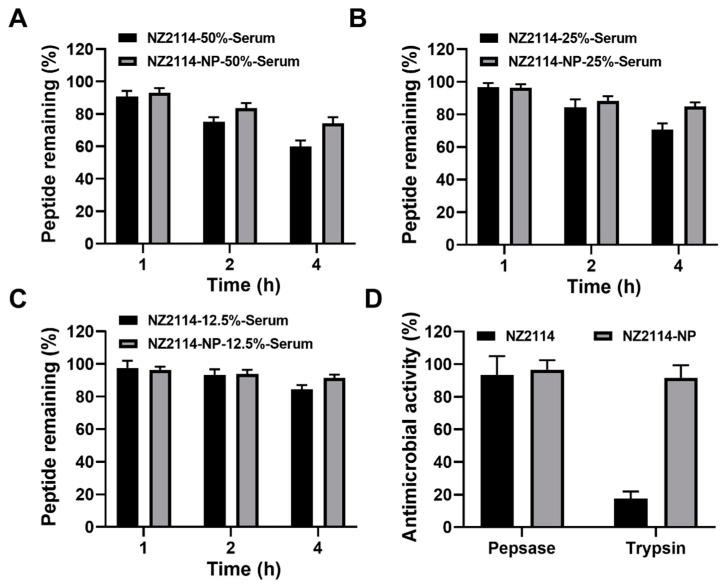
The stability of NZ2114 and NZ2114-NPs under different physiological conditions. (**A**–**C**) Peptide retention of NZ2114 and NZ2114-NPs at serum concentrations of 50%, 25% and 12.5%, respectively. (**D**) Effects of pepsin and trypsin on the antimicrobial activity of NZ2114 and NZ2114-NPs against *S. epidermidis* G4. NZ2114-NPs: PLGA-encapsulated NZ2114 (formulation-13). Results are given as mean ± SD (*n* = 3).

The stability of NZ2114 and NZ2114-NPs in different concentrations of serum was assessed by RP-HPLC. The results showed that after incubation in 50% serum for 1–4 h, the retention of NZ2114 and NZ2114-NPs was 90.59–59.83% and 93.06–74.26%, respectively (Figure 3A). After incubation in 25% serum for 1–4 h, the retention of NZ2114 and NZ2114-NPs was 96.85–65.69% and 97.54–84.83%, respectively (Figure 3B). After incubation in 12.5% serum for 1–4 h, the retention rates were 97.64–84.34% and 96.33–91.47%, respectively (Figure 3C). Compared with the free peptide NZ2114, PLGA encapsulation increased the retention of NZ2114 in 50%, 25% and 12.5% serum by 24.12%, 20.01%, and 8.45%, respectively.

### 2.5. Enzymatic Resistance of NZ2114-NPs

The ability of PLGA encapsulation to protect NZ2114 against enzymatic degradation was determined by zone inhibition assay. As shown in Figure 3D, after incubation with pepsin for 2 h, NZ2114 and NZ2114-NPs retained 93% and 97% antimicrobial activity against *S. epidermidis* G4, respectively, indicating that NZ2114-NPs exhibited better pepsin resistance. While after incubation with trypsin for 2 h, the retention of antibacterial activity of NZ2114-NPs against *S. epidermidis* G4 was 92%, which was 4.24-fold higher than that of NZ2114 (18%), indicating that the encapsulation of PLGA could effectively protect NZ2114 from degradation by trypsin.

### 2.6. MIC and MBC Determination

The MIC and MBC of NZ2114 and NZ2114-NPs against *S. epidermidis* are shown in Table 3. The MIC of NZ2114 against *S. epidermidis* ranges from 2 to 8 μg/mL, and that of NZ2114-NPs ranges from 4 to 8 μg/mL, and the unloaded NPs had no antimicrobial activity. There was essentially no difference in the MIC of NZ2114 and NZ2114-NPs against *S. epidermidis*, indicating that the encapsulation process (organic solvent, shear, surfactant) had little effect on the bioactivity of NZ2114 and PLGA preparation retained the bioactivity of the peptide. Meanwhile, the MBC results showed that the MBC of NZ2114 against *S. epidermidis* was two to eight times higher than the MIC, and that of NZ2114-NPs was two to four times higher than the MIC; especially, the MBC of them against *S. epidermidis* ATCC 35984 and *S. epidermidis* G11 was relatively higher, which may be due to the strong ability of these two strains to form biofilms.

### 2.7. Effect of NZ2114-NPs on Bacteria in Biofilms

**Figure 4:**
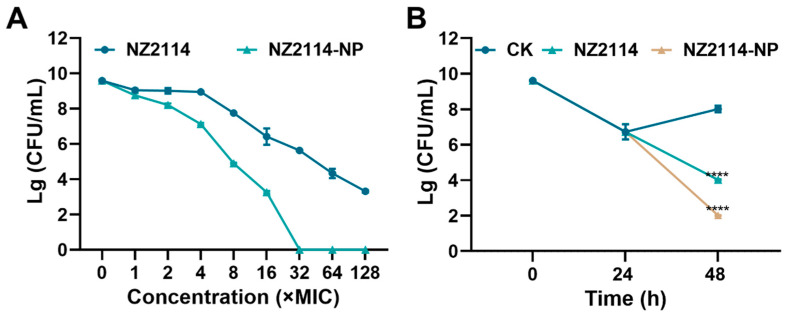
Effect of NZ2114 and NZ2114-NPs on *S. epidermidis* G4 biofilm bacteria. (**A**) Bactericidal activity of NZ2114 and NZ2114-NPs against established biofilms of *S. epidermidis* G4; (**B**) bactericidal activity of NZ2114 and NZ2114-NPs against persisters from the biofilm. NZ2114-NP: PLGA-encapsulated NZ2114 (formulation-13). CK: PBS group. Results were given as mean ± SD (*n* = 3). ****: *p* < 0.0001.

As shown in Figure 4A, both NZ2114 and NZ2114-NPs reduced the number of *S. epidermidis* G4 in biofilms. After 1-128× MIC NZ2114 treatment, the colony number of bacteria decreased by 0.578 to 6.27 lg. The colony number of *S. epidermidis* G4 decreased by 0.839–9.58 lg, and NZ214-NPs (32× MIC) showed 100% bactericidal effect against *S. epidermidis* in mature biofilms. In conclusion, the encapsulation of NZ2114 by PLGA significantly enhanced the bactericidal effect of NZ2114 against *S. epidermidis* in biofilms compared to free NZ2114.

### 2.8. Effect of NZ2114-NPs on Biofilm Persister

To further assess the effect of NZ2114-NPs on persister bacteria in mature biofilms, vancomycin (100× MIC) was added to induce biofilm persister [16]. As shown in Figure 4B, after incubation with vancomycin (100× MIC) for 24 h, the number of viable bacteria in the biofilm was 5.3 × 10^6^ CFU/mL. The number of persistent bacteria decreased to 1.04 × 10^4^ CFU/mL after treatment with NZ2114 (16× MIC) for 24 h, and the elimination rate of persistent bacteria was 49.8%. The elimination rate of the retention bacteria in the NZ2114-NPs (16× MIC) treatment group reached 99%, which was superior to that of the NZ2114 group. Compared to the untreated group, the difference was highly significant (*p* < 0.0001).

### 2.9. Observation of Biofilms by Confocal Laser Scanning Microscopy (CLSM)

**Figure 5:**
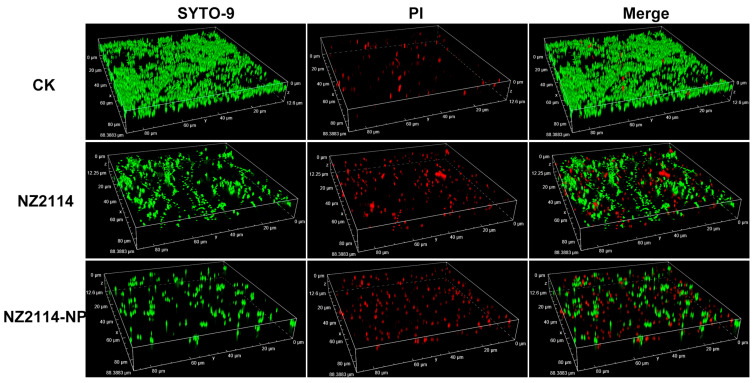
Effects of NZ2114 and NZ2114-NPs on *S. epidermidis* G4 biofilms by CLSM. NZ2114-NP: PLGA-encapsulated NZ2114 (formulation-13). CK: PBS group, SYTO (Green): live bacteria, PI (Red): dead bacteria.

The elimination and inhibition of NZ214-NPs against *S. epidermidis* G4 biofilms and internal bacteria were further verified by CLSM using SYTO-9 and PI. As shown in Figure 5, in the untreated group (CK), *S. epidermidis* G4 formed a biofilm with a thickness of 12.6 µm and more than 95% were live cells (green) in the field of view (12.6 μm), whereas only scattered single cells were observed in the field of view and the flocculent biofilm almost disappeared after the treatments of NZ2114 and NZ2114-NPs (16× MIC). In contrast, the NZ2114-NPs were more effective than NZ2114, which was consistent with the above results of colony counting.

## 3. Discussion

Previous studies have shown that the tunable surface functionality of nanomaterials provides scope for fine-tuned design, offering an ‘out-of-the-box’ approach to biofilm infection [29,30,31]. Building on this, this study aims to improve the bioavailability and delivery efficiency of NZ2114 in biofilms by encapsulation. Among various materials, PLGA, as a biocompatible and degradable copolymer, is expected to protect NZ2114 from binding to EPS components (DNA or polysaccharides) when used as a drug delivery carrier. Its biphasic release behavior perfectly matches the drug concentration requirements of biofilm eradication [32]. PLGA was selected as the ideal encapsulation material of NZ2114 for orthogonal design to screen formulations with high drug-loading rates, high release rates, and uniform distribution.

Particle size and size distribution (PDI) are important parameters in the development of nanomedicines, which further affects the drug loading, drug release and drug stabilization within the nanoparticles [33]. To study the effect of formulation on nanoparticles, nanoparticle particle size and size distribution were determined. The results are shown in Table 2. Overall, the particle size distribution (186–412 nm) of PLGA (50:50, dL: 0.3) was larger than that of the corresponding PLGA (50:50, dL: 0.14), which is in agreement with the results of a previous study: the particle size increases with the molecular weight of the polymer, which may be due to the fact that when the molecular weight of the polymer increases, the organic phase becomes more viscous, which leads to an increase in the viscous forces that resist the disintegration of the droplets through sonication. These forces are opposite to the shear stress in the organic phase, and the final size of the particles depends on the net shear stress available for droplet break-up, making it difficult to obtain small emulsion droplets at the same mixing rate [34]. However, PLGA (75:25) did not show the same pattern, which could be related to the influence of the corresponding surfactant on it. Meanwhile, ζ potential is one of the key factors affecting the stability of particles in dispersions, and the results showed that the ζ potential increased with the increase in drug loading, which is in agreement with the previous studies and may be due to the saturation of peptides in the oil–water interface, which makes them not only confined to the core of the nanoparticles but mainly localized on the surface of the nanoparticles, resulting in a significant increase in the zeta potential of the nanoparticles [23].

The molecular weight and L:G ratio of the PLGA can affect the overall pore size of the particles by influencing the particle formation process (aggregation, shell formation, and solidification), which in turn determines the drug-loading capacity, initial rupture and subsequent drug release kinetics [27]. The aim of this study was to screen the optimal PLGA encapsulation formulation by orthogonal design. As shown in Table 2 and Figure 1A–D, the corresponding cumulative release rates of PLGA (50:50, dL-0.14; 50:50, dL-0.3; 75:25, dL-0.16; and 75:25, dL-0.3) were 63.38–91.09%, 47.62–78.33%, 67.75–93.55%, and 59.03–84.84%, respectively. In general, the cumulative release rate of NZ2114 in PLGA (50:50) was lower than that of PLGA (75:25). This is partly attributed to the fact that an increase in the concentration of polylactic acid slows down degradation due to the presence of hydrophobic methyl groups, leading to slower water uptake and diffusion and thus reducing drug release [35,36].

As a stabilizer, surfactant PVA, with one end adsorbed on the surface of particles and the other end in solution, shielding the particles by bridging hydroxyl groups, greatly reducing the attraction between particles and thus making the particles in a highly dispersed state, has been used as a PLGA-encapsulated stabilizer [23,37]. In PLGA encapsulation, the molecular weight and concentration of PVA affect the viscosity of the external aqueous phase and thus the rate of diffusion of acetone from the polymer mechanism into the external aqueous phase: a decrease in PVA viscosity reduces the viscosity of the external aqueous phase, which promotes rapid diffusion of the solvent and consequently the encapsulation of the drug in the polymer and the formation of small nanoparticles [38]. As shown in the results, the particle size distribution of the PVA 205 encapsulation product was 165.05–186.69 nm (Table 2), and the cumulative release rate was 61.55–91.09%, which was better than that of the other encapsulation products (Figure 1A–D).

The interaction between the drug, PLGA, PVA stabilizer, and solvent affects the properties of the particles in an unpredictable and non-linear manner, and therefore each drug must be tailored to a specific PLGA type and stabilizer class based on its unique physicochemical properties. In this process, PLGA drug encapsulation requires a comprehensive balance of encapsulation and release rates to maximize drug loading and minimize particle size [39,40]. Based on this, the present study was designed to obtain the optimal formulation 13 consisting of PLGA (75:25, dL: 0.16) and PVA 205 encapsulated with a drug-loading rate of 5%. It is worth noting that it is generally believed that the initial abrupt release of PLGA is due to the high porosity of the particles, whereas the SEM results (Figure 1) showed that the surface of the NZ2114-NPs was smooth with no visible pores, which seems to be inconsistent with the results of Figure 1D. We speculate that the initial pore size of these particles may be too small to be detected by SEM, or that SEM is the initial state of NZ2114-NPs in solution rather than after burst release in PBS (12 h), and the degradation of the polymer into pores that may occur during this process is not captured, thus causing inconsistency with the release results, but this needs further verification [41,42,43]. 

Safety is a prerequisite for drug development. To investigate whether the encapsulation of PLGA could improve the cell selectivity of NZ2114, the cytotoxicity of NZ2114-NPs against HaCaT cells was determined. The results showed that PLGA encapsulation effectively improved the cytotoxicity of NZ2114 on HaCaT cells (Figure 2B), which was consistent with the previous study showing that the encapsulation of SAAP-148 by PLGA reduces the cytotoxicity of SAAP-148 to primary skin fibroblasts by 24–41-fold [26]. Stability is an important consideration in the development of peptide drugs. In this study, NZ2114 retained only 59.83% of the monomeric peptide after 4 h incubation in 50% serum and only 18% after incubation in trypsin, which is consistent with previous studies, whereas its tolerance to serum protease and trypsin was increased by 224.12% and 424%, respectively (Figure 3) [28], suggesting that PLGA encapsulation can be an effective strategy to improve the protease tolerance of AMPs. This may be due to the spatial barrier provided by the hydrophobic PLGA blocking enzyme interactions [44,45].

Nanoparticles make a very promising treatment modality for biofilms; the delivery system protects the peptide from pH and enzymatic degradation in complex biofilms while increasing the penetration efficiency of the AMP into the biofilm due to the size effect of the nanoparticles [29,46]. PLGA-encapsulated ciprofloxacin has been reported to be used for the treatment of endodontic infections caused by *E. faecalis*, and the results of clinical trials have shown its success in eradicating bacteria from biofilms [47]. The results of this study show that the bactericidal rate of NZ2114-NPs (32× MIC) against *S. epidermidis* in mature biofilm reached 100%, and the bactericidal rate of NZ2114-NPs against biofilm persister was 99%, which was significantly higher than that of the free peptide NZ2114 (Figure 4). This may be attributed to the particle property of NZ2114-NP, which has a particle size of 178 nm (<350 nm) and can diffuse through the pores of the biofilm for diffusion [29]; meanwhile, the positive electrical charge (ζ potential: +4.78) of NZ2114-NPs can disrupt the biofilm to promote the penetration of particles into the biofilm, thus improving the antimicrobial efficiency [48]. This experiment further demonstrated the promising application of nanoparticles in the field of biofilm removal. However, the in vivo bioavailability and efficacy of NZ2114-NPs need to be further evaluated.

## 4. Materials and Methods

### 4.1. Materials

PLGA 75:25 (intrinsic viscosity 0.16 dL/g, average Mw 7000–20,000 Da), PLGA 75:25 (intrinsic viscosity 0.3 dL/g, average Mw 20,000–50,000 Da), PLGA 50:50 (intrinsic viscosity 0.14 dL/g, average Mw 7000–20,000 Da) and PLGA 50:50 (intrinsic viscosity 0.3 dL/g, average Mw 20,000−50,000 Da) were purchased from the Shandong Academy of Pharmaceutical Sciences (Jinan, China). PVA224 (Lot: D218362), PVA117 (Lot: K2013111), PVA1788 (Lot: I2111237), and PVA205 (Lot: H172877) were purchased from Aladdin Co., Ltd. (Beijing, China). The Cell-Counting Kit (CCK-8, Lot:C8216990) was purchased from Yeasen Biotechnology Co., Ltd. (Shanghai, China). High-performance liquid chromatography (HPLC) grade acetonitrile, dichloromethane and 96% ethanol were purchased from Thermo-Fisher Scientific (Shanghai, China). NZ2114 (with purity over 90%) was prepared as described in our previous study [49]. *S. epidermidis* G4 and G11 were isolated by China Agricultural University, and *S. epidermidis* ATCC 35984 and ATCC12228 were purchased from the American Type Culture Collection (ATCC, Manassas, VA, USA).

### 4.2. Preparation of Nanoparticles

The formulation of NZ2114 nanoparticles was optimized by an orthogonal test. PLGA type (A), drug-loading rate (B), and PVA type (C) were used as influencing factors for orthogonal design, and four levels were selected for each factor. The settings levels of each factor are shown in Table 4 and Table 5. NZ2114 nanoparticles were prepared by the water–oil–water-in-water (W/O/W) double-emulsion solvent evaporation method. The specific scheme follows. (1) Primary emulsion preparation: 500 μL of NZ2114 stock solution with different concentrations was added to a 500 μL solution of 60 mg/mL PLGA dichloromethyl, and the mixture was homogenized to form a primary emulsion by Ultrasonic Homogenizer (SCIENTZ-IID) at 60% amplitude for 2 min. (2) Preparation of double emulsion: 1 mL of 2% PVA aqueous solution was added to the primary emulsion and mixed, and two rounds of emulsion were obtained using the ultrasound scheme mentioned above. (3) Nanoparticle preparation: 5 mL PVA aqueous solution was added to the above double emulsion, and the organic solvent was removed by rotary evaporation at room temperature for 2 h, followed by centrifugation (22,000× *g*, 12 min), washing with ultrapure water twice, and freeze-drying after ultrasonic for 24 h to obtain nanoparticles, and then it was placed at −20 °C for reserve [23].

### 4.3. Characterization of NZ2114-NP

#### 4.3.1. Particle Distribution

The particle size and polydispersity index of the samples were determined by dynamic light scattering (DLS). NZ2114-NP samples were dispersed in ultrapure water and diluted 50-fold for determination. The DLS and polydispersity index (PDI) were measured by a Zetasizer Nano ZS (Malvern Instruments, Malvern, UK) at a scattering angle of 173 and 25 °C.

#### 4.3.2. Zeta Potential

The electrophoretic mobility of samples from the same batch was determined by laser Doppler electrophoresis using a Zetasizer Nano ZS and converted to ζ potential by Smoluchowski’s equation with three replicates per batch.

#### 4.3.3. Scanning Electron Microscopy

An electron microscope was used to observe the surface morphology of the nanoparticles. The freeze-dried nanoparticles obtained were redissolved in ultrapure water and sonicated for 5 min to uniformly distribute the sample, after which a 4 μL drop of the sample was taken on the front of the wafer, dried, sprayed with gold, and imaged through a scanning electron microscope (Hitachi SU8000, Tokyo, Japan) at an accelerating voltage of 5 KV [50].

#### 4.3.4. RP-HPLC Analysis of NZ2114

Quantification was performed using reversed-phase high performance liquid chromatography (RP-HPLC) (Agilent 1260 Infinity, Santa Clara, CA, USA) with UV absorption detector composition. Stationary phase: Boston Green ODs-AQ (250 mm × 4.6 mm, 5 μm, 120 Å), mobile phase: 0.1% TFA acetonitrile solution; B: 0.1% TFA aqueous solution. The gradient elution conditions were as follows: 0–16 min, 20–45% of mobile phase A; flow rate of 1.0 mL/min; detection wavelength: 280 nm; injection volume: 50 μL. A calibration curve was established under the experimental conditions (*n* = 3), and the calibration curves showed a linear range of 8–512 μg/mL with correlation coefficients of R^2^ greater than 0.999. The drug concentration in the samples was calculated [51,52]. 

#### 4.3.5. Drug Loading and Encapsulation Efficiency

In order to determine the maximum loading capacity of PLGA, the encapsulation scheme with different drug-loading capacity was designed for the encapsulation of NZ2114, and the drug-loading rate (DL) was calculated according to Equation (1).

(1) $$\mathrm{Drug}\;\mathrm{loading}\;\mathrm{rate}\;\left(\%\right)=\frac{\mathrm{NZ}2114\;\mathrm{mass}}{\mathrm{PLGA}\;\mathrm{mass}}\times 100\%\;$$

Meanwhile, the encapsulation rate of PLGA was determined by an indirect method as follows: free NZ2114 in the supernatant obtained by centrifugation and filtration in 4.2 was quantified by RP-HPLC, and the encapsulation efficiency (EE) was calculated by the following Equation (2) [44].

(2) $$\;\mathrm{Encapsulation}\;\mathrm{efficiency}\left(\mathrm{EE}\%\right)=\frac{(Th\mathrm{eoretical}\;\mathrm{NZ}2114\;\mathrm{loading}\;-\;\mathrm{free}\;\mathrm{NZ}2114)}{\mathrm{Theoretical}\;\mathrm{NZ}2114\;\mathrm{loading}}\times 100\%$$

#### 4.3.6. Release Rate

The nanoparticles obtained by freeze-drying were transferred to a 50 mL volumetric flask, then redispersed by adding 10 mL PBS buffer, and then incubated in a linear shaking water bath at 37 °C (100 rpm). Then, 1 mL of the suspended sample was removed at a predetermined time and replenished with 1 mL of PBS; the suspension was centrifuged at 12,000 rpm for 10 min and the supernatant was collected for quantitative analysis by RP-HPLC. Release assays were performed in triplicate [23,53].

### 4.4. Safety and Stability

#### 4.4.1. Hemolysis Analysis

Mouse blood erythrocytes were used to assess the hemolysis of NZ2114 nanoparticles. Mouse erythrocytes were diluted to 8% with 0.9% NaCl, and then an equal volume of different concentrations of nanoparticles was added and mixed, and the samples were incubated at 37 °C for 1 h. After incubation, samples were centrifuged at 1500 rpm for 5 min at 4 °C, and the supernatant was collected and transferred to a 96-well plate for determination. Then, 0.9% NaCl solution and 0.1% tritone X-100 were used as the negative control (A_0_) and positive control (A_100_), respectively. Hemolysis was calculated according to Equation (3) [9].

(3) $$\mathrm{Hemolysis}\;\left(\%\right)=\frac{A_{NP}-\;A_{0}}{A_{100}-\;A_{0}}\times 100\%\;$$

#### 4.4.2. Cytotoxicity

The cytotoxicity of HaCaT was determined using CCK-8. The method was performed as follows. (1) Cell culture: HaCaT cells at a density of 2.5 × 10^5^ cells/mL were inoculated into 96-well cell culture plates and incubated for 24 h (37 °C, 5% CO_2_, saturated humidity conditions); (2) drug incubation: after removing the medium, an equal volume of NZ2114 nanoparticles was added and incubated for 12 h; (3) CCK-8 incubation: the supernatant was removed, CCK-8 (1/10 dilution) was added and incubated for 2 h, and the absorbance value at 450 nm was detected by a Microplate Reader; (4) cell survival rate was calculated according to Equation (4) [9,54].

(4) $$\mathrm{Cell}\;\mathrm{survival}\;\mathrm{rate}\;\left(\%\right)=\frac{A_{NP}}{A_{PBS}}\times 100\%$$

#### 4.4.3. Serum Stability

To assess the stability of NZ2114-NPs under physiological conditions, nanoparticles obtained by freeze-drying were resuspended in 1.5 mL of mouse serum at final concentrations of 12.5%, 25% and 50%, incubated at 37 °C for 0, 1, 2, and 4 h and quantified by RP-HPLC [53,55].

#### 4.4.4. Enzymatic Resistance of NZ2114-NP

The enzymatic stability of NZ2114 nanoparticles was determined by inhibition zone assay [28]. Free or encapsulated NZ2114 was incubated with trypsin or pepsin for 2 h at 37 °C to determine protease stability. Untreated peptide and buffer alone were used as positive and negative controls, respectively. All assays were performed in triplicate.

### 4.5. In Vitro Antimicrobial Activity

#### 4.5.1. MIC and MBC Determination

The MIC values of NZ2114 nanoparticles were assessed using the broth microdilution method [52]. Briefly, *S. epidermidis* in the logarithmic growth phase (1 × 10^5^ CFU/mL) was incubated with different concentrations of samples for 16 to 20 h at 37 °C. The MIC value was defined as the lowest drug concentration at which there was no visible bacterial growth, and the MBC value was defined as the lowest drug concentration at which 99% of the bacteria were killed.

#### 4.5.2. Effect of NZ2114-NPs on Bacteria in Biofilms

The effect of NZ2114 nanoparticles on the bacteria in the biofilm was determined according to Yang’s method [56]. Briefly, *S. epidermidis* G4 (1 × 10^8^ CFU/mL) was incubated at 37 °C for 24 h, the supernatant was removed and washed with PBS, and different concentrations of NZ2114 or NZ2114-NPs were added and incubated for 12 h, and then colonies were counted.

#### 4.5.3. Effect of NZ2114-NPs on Biofilm Persister

The antibacterial effect of NZ2114 nanoparticles on the persistent bacteria in the biofilm was determined according to the previous study [56]. Briefly, *S. epidermidis* G4 (1 × 10^8^ CFU/mL) was incubated at 37 °C for 24 h, 100× MIC vancomycin was added for 24 h to remove planktonic bacteria, NZ2114 or NZ2114-NPs (16× MIC) were added and incubated for 24 h, and then colonies were counted.

#### 4.5.4. Observation of Biofilms by Confocal Laser Scanning Microscopy

The effect of NZ2114 on the biofilm and internal bacteria was observed according to the previous study [16]. After NZ2114-NP treatment, samples were incubated with PI and SYTO9 (LIVE/DEAD BacLight Bacterial Viability Kit, ThermoFisher, Shanghai, China) for 15 min and observed by CLSM (Zeiss LSM880, Carl Zeiss, Oberkochen, Germany).

### 4.6. Statistical Analysis

Data were statistically analyzed using GraphPad Prism software v9.0 (GraphPad Software, San Diego, CA, USA) and are expressed as mean ± standard deviation (SD). Univariate ANOVA and Tukey multiple comparisons were used to analyze the statistical significance of the differences between groups.

## 5. Conclusions

In summary, the nanodelivery system of NZ2114-NPs achieved the following: (i) favorable physicochemical properties: particle size: 178.11 ± 5.23 nm, uniform distribution, drug loading: 4.074 ± 0.37%, encapsulation rate: 81.46 ± 7.42%, biphasic release mode: initial burst of sudden release followed by sustained release, cumulative release rate: 67.75% (120 h); (ii) excellent safety and stability: hemolysis of mouse erythrocytes and cytotoxicity of HaCaT was less than 5%, peptide retention in 50% of serum was increased by 24.12% for NZ2114-NPs, and peptide retention in 50% serum was increased by 24.12% for NZ2114-NPs), and the tolerance to trypsin was increased by 4.24 times; (iii) excellent antimicrobial activity: MIC against *S. epidermidis* was as low as 4–8 μg/mL, and the bactericidal rate of NZ2114-NPs against *S. epidermidis* G4 in mature biofilm was 9–100%, which was superior to that of NZ2114 (6–65%), the bactericidal activity of NZ2114-NPs against *S. epidermidis* G4 persister was 99%, which was better than that of NZ2114 (49.8%); this may be attributed to the sustained release effect of PLGA to protect NZ2114 from interference with the complex pH and enzyme environment within the biofilm. In conclusion, our data provide evidence that PVA-engineered PLGA nanoparticles can be used as valuable nanocarriers for the treatment of biofilm caused by *S. epidermidis*.

## Figures and Tables

**Table 1:** Structure and properties of NZ2114.

Antimicrobial Peptide	NZ2114
Sequence (amino acid)	GFGCNGPWNEDDLRCHNHCKSIKGYKGGYCAKGGFVCKCY
Cyclic site	Cys4-Cys30, Cys15-Cys37, Cys19-Cys39
Structure type	CSαβ
Molecular weight (Da)	4417.03
Number of amino acids	40
Charge number	+3
Theoretical PI	8.62
Hydrophobicity	0.35
Grand average of hydropathicity (GRAVY)	−0.672

**Table 2:** Physicochemical characterization of NZ2114-loaded PLGA nanoparticles (mean ± SD, *n* = 3).

Formulation	Theoretical Loading (%)	PLGA Type (L/G, dL/g)	PVA Type	Size (nm)	PDI (Polydispersity Index)	ζ-Potential (Zeta Potential, mV)	Encapsulation Rate (EE %)	Actual Loading Rate (%)
1	0.625	75:25 (0.16)	224	378.50 ± 6.08	0.182 ± 0.01	−12.6 ± 0.21	91.5 ± 1.44	0.57 ± 0.01
2	0.625	75:25 (0.30)	117	224.29 ± 3.10	0.225 ± 0.03	−18.2 ± 0.96	92.48 ± 1.72	0.58 ± 0.01
3	0.625	50:50 (0.30)	1788	243.67 ± 2.12	0.096 ± 0.01	−22.3 ± 0.52	96.18 ± 3.01	0.60 ± 0.02
4	0.625	50:50 (0.14)	205	165.05 ± 2.21	0.045 ± 0.01	−17.6 ± 0.52	94.50 ± 3.77	0.59 ± 0.02
5	1.25	75:25 (0.16)	117	365.45 ± 10.05	0.325 ± 0.02	−9.56 ± 0.25	89.91 ± 1.95	1.12 ± 0.02
6	1.25	75:25 (0.30)	224	226.94 ± 3.02	0.115 ± 0.01	−12.13 ± 0.56	90.00 ± 2.38	1.13 ± 0.03
7	1.25	50:50 (0.30)	205	186.69 ± 1.05	0.156 ± 0.02	−11.56 ± 0.62	94.48 ± 3.72	1.18 ± 0.05
8	1.25	50:50 (0.14)	1788	281.81 ± 2.05	0.083 ± 0.01	−10.28 ± 0.91	92.06 ± 3.95	1.15 ± 0.05
9	2.5	75:25 (0.16)	1788	487.67 ± 6.02	0.232 ± 0.02	−3.56 ± 0.52	80.30 ± 3.64	2.01 ± 0.09
10	2.5	75:25 (0.30)	205	169.67 ± 3.56	0.125 ± 0.01	−5.93 ± 0.24	83.70 ± 2.18	2.09 ± 0.05
11	2.5	50:50 (0.30)	224	321.77 ± 2.31	0.240 ± 0.05	2.56 ± 0.12	87.65 ± 5.70	2.19 ± 0.14
12	2.5	50:50 (0.14)	117	346.67 ± 7.32	0.135 ± 0.01	1.32 ± 0.36	82.26 ± 3.57	2.06 ± 0.09
13	5.0	75:25 (0.16)	205	178.11 ± 5.23	0.108 ± 0.10	4.78 ± 0.67	81.46 ± 7.42	4.07 ± 0.37
14	5.0	75:25 (0.30)	1788	244.72 ± 1.65	0.250 ± 0.01	7.56 ± 0.24	75.87 ± 1.15	3.79 ± 0.06
15	5.0	50:50 (0.30)	117	412.40 ± 16.58	0.241 ± 0.01	4.32 ± 0.57	76.36 ± 7.42	3.82 ± 0.37
16	5.0	50:50 (0.14)	224	237.19 ± 7.54	0.185 ± 0.01	8.24 ± 0.45	81.63 ± 10.38	4.08 ± 0.52

**Table 3:** Antimicrobial activity of NZ2114 and NZ2114-NPs against *Staphylococcus epidermidis*.

Strains		MIC (μg/mL)		MBC (μg/mL)	
	Blank-NP	NZ2114	NZ2114-NP	NZ2114	NZ2114-NP
*Staphylococcus epidermidis* ATCC 35984	>512	8	8	16	16
*S. epidermidis* ATCC 12228	>512	2	4	4	8
*S. epidermidis* G4	>512	2	4	16	8
*S. epidermidis* G11	>512	4	8	32	32

**Table 4:** Orthogonal test factor level.

Value Level	Factor Level		
	A: NZ2114 Concentration (*w*/*v* %)	B: PLGA Type (L:G, dL/g)	C: PVA Type
1	0.625	75:25 (0.16)	224
2	1.25	75:25 (0.30)	117
3	2.50	50:50 (0.30)	1788
4	5.00	50:50 (0.14)	205

**Table 5:** Orthogonal test design.

Formula No.	A	B	C
1	1	1	1
2	1	2	2
3	1	3	3
4	1	4	4
5	2	1	2
6	2	2	1
7	2	3	4
8	2	4	3
9	3	1	3
10	3	2	4
11	3	3	1
12	3	4	2
13	4	1	4
14	4	2	3
15	4	3	2
16	4	4	1

## Data Availability

The original contributions presented in the study are included in the article; further inquiries can be directed to the corresponding author(s).

## References

[1] Holmes A.H., Moore L.S., Sundsfjord A., Steinbakk M., Regmi S., Karkey A., Guerin P.J., Piddock L.J. (2016). Understanding the mechanisms and drivers of antimicrobial resistance. Lancet.

[2] Dalhoff A. (2012). Resistance surveillance studies: A multifaceted problem—The fluoroquinolone example. Infection.

[3] Han C., Romero N., Fischer S., Dookran J., Doiron A.L. (2016). Recent developments in the use of nanoparticles for treatment of biofilms. Nanotechnol. Rev..

[4] Leung S.S.Y., Chan H.-K. (2022). Emerging antibiotic alternatives: From antimicrobial peptides to bacteriophage therapies. Adv. Drug Deliv. Rev..

[5] Murray C.J.L., Ikuta K.S., Sharara F., Swetschinski L., Aguilar G.R., Gray A., Han C., Bisignano C., Rao P., Wool E. (2022). Global burden of bacterial antimicrobial resistance in 2019: A systematic analysis. Lancet.

[6] De Oliveira D.M.P., Forde B.M., Kidd T.J., Harris P.N.A., Schembri M.A., Beatson S.A., Paterson D.L., Walker M.J. (2022). Antimicrobial resistance in ESKAPE pathogens. Clin. Microbiol. Rev..

[7] Lazzaro B.P., Zasloff M., Rolff J. (2020). Antimicrobial peptides: Application informed by evolution. Science.

[8] Zhang Q.-Y., Yan Z.-B., Meng Y.-M., Hong X.-Y., Shao G., Ma J.-J., Cheng X.-R., Liu J., Kang J., Fu C.-Y. (2021). Antimicrobial peptides: Mechanism of action, activity and clinical potential. Mil. Med. Res..

[9] Hao Y., Teng D., Mao R., Yang N., Wang J. (2023). Site mutation improves the expression and antimicrobial properties of fungal defense. Antibiotics.

[10] Xuan J., Feng W., Wang J., Wang R., Zhang B., Bo L., Chen Z.-S., Yang H., Sun L. (2023). Antimicrobial peptides for combating drug-resistant bacterial infections. Drug Resist. Update.

[11] Xu S., Tan P., Tang Q., Wang T., Ding Y., Fu H., Zhang Y., Zhou C., Song M., Tang Q. (2023). Enhancing the stability of antimicrobial peptides: From design strategies to applications. Chem. Eng. J..

[12] Ewles M., Goodwin L. (2011). Bioanalytical approaches to analyzing peptides and proteins by LC--MS/MS. Bioanalysis.

[13] Heng W., Wu H., Ciofu O., Song Z., Hiby N. (2012). In vivo pharmacokinetics/pharmacodynamics of colistin and imipenem in *Pseudomonas aeruginosa* biofilm infection. Antimicrob. Agents Chemother..

[14] Jiang Y., Chen Y., Song Z., Tan Z., Cheng J. (2021). Recent advances in design of antimicrobial peptides and polypeptides toward clinical translation. Adv. Drug Deliv. Rev..

[15] Jin Y., Yang N., Teng D., Hao Y., Mao R., Wang J. (2023). Molecular modification of kex2 P1’ site enhances expression and druggability of fungal defensin. Antibiotics.

[16] Yang N., Zhang Q., Mao R., Hao Y., Ma X., Teng D., Fan H., Wang J. (2022). Effect of NZ2114 against *Streptococcus dysgalactiae* biofilms and its application in murine mastitis model. Front. Microbiol..

[17] Wang C., Hong T., Cui P., Wang J., Xia J. (2021). Antimicrobial peptides towards clinical application: Delivery and formulation. Adv. Drug Deliv. Rev..

[18] Imperlini E., Massaro F., Buonocore F. (2023). Antimicrobial peptides against bacterial pathogens: Innovative delivery nanosystems for pharmaceutical applications. Antibiotics.

[19] Fonte P., Araújo F., Silva C., Pereira C., Sarmento B. (2015). Polymer-based nanoparticles for oral insulin delivery: Revisited approaches. Biotechnol. Adv..

[20] Manchanda R., Fernandez-Fernandez A., Nagesetti A., McGoron A.J. (2010). Preparation and characterization of a polymeric (PLGA) nanoparticulate drug delivery system with simultaneous incorporation of chemotherapeutic and thermo-optical agents. Colloids Surf. B Biointerfaces.

[21] Martins C., Sousa F., Araújo F., Sarmento B. (2017). Functionalizing PLGA and PLGA derivatives for drug delivery and tissue regeneration applications. Adv. Healthc. Mater..

[22] El-Hammadi M.M., Arias J.L. (2022). Recent advances in the surface functionalization of PLGA-based nanomedicines. Nanomaterials.

[23] Water J.J., Smart S., Franzyk H., Foged C., Nielsen H.M. (2015). Nanoparticle-mediated delivery of the antimicrobial peptide plectasin against *Staphylococcus aureus* in infected epithelial cells. Eur. J. Pharm. Biopharm..

[24] D’angelo I., Casciaro B., Miro A., Quaglia F., Mangoni M.L., Ungaro F. (2015). Overcoming barriers in *Pseudomonas aeruginosa* lung infections: Engineered nanoparticles for local delivery of a cationic antimicrobial peptide. Colloids Surf. B Biointerfaces.

[25] Casciaro B., d’Angelo I., Zhang X., Loffredo M.R., Conte G., Cappiello F., Quaglia F., Di Y.-P.P., Ungaro F., Mangoni M.L. (2019). Poly(lactide-co-glycolide) nanoparticles for prolonged therapeutic efficacy of esculentin-1a-derived antimicrobial peptides against *Pseudomonas aeruginosa* lung infection: In vitro and in vivo studies. Biomacromolecules.

[26] Ali M., van Gent M.E., de Waal A.M., van Doodewaerd B.R., Bos E., Koning R.I., Cordfunke R.A., Drijfhout J.W., Nibbering P.H. (2023). Physical and functional characterization of PLGA nanoparticles containing the antimicrobial peptide SAAP-148. Int. J. Mol. Sci..

[27] Park K., Skidmore S., Hadar J., Garner J., Wang Y. (2019). Injectable, long-acting PLGA formulations: Analyzing PLGA and understanding microparticle formation. J. Control. Release.

[28] Liu H., Yang N., Teng D., Mao R., Hao Y., Ma X., Wang J. (2021). Design and pharmacodynamics of recombinant fungus defensin NZL with improved activity against *Staphylococcus hyicus* in vitro and in vivo. Int. J. Mol. Sci..

[29] Makabenta J.M.V., Nabawy A., Li C.H., Schmidt-Malan S., Rotello V.M. (2020). Nanomaterial-based therapeutics for antibiotic-resistant bacterial infections. Nat. Rev. Microbiol..

[30] Al-Wrafy F.A., Al-Gheethi A.A., Ponnusamy S.K., Noman E.A., Fattah S.A. (2021). Nanoparticles approach to eradicate bacterial biofilm-related infections: A critical review. Chemosphere.

[31] Liu Y., Shi L., Su L., van der Mei H.C., Jutte P.C., Ren Y., Busscher H.J. (2019). Nanotechnology-based antimicrobials and delivery systems for biofilm-infection control. Chem. Soc. Rev..

[32] Mundargi R.C., Babu V.R., Rangaswamy V., Patel P., Aminabhavi T.M. (2008). Nano/micro technologies for delivering macromolecular therapeutics using poly(D,L-lactide-co-glycolide) and its derivatives. J. Control. Release.

[33] Akl M.A., Kartal-Hodzic A., Oksanen T., Ismael H.R., Afouna M.M., Yliperttula M., Samy A.M., Viitala T. (2016). Factorial design formulation optimization and in vitro characterization of curcumin-loaded PLGA nanoparticles for colon delivery. J. Drug Deliv. Sci. Technol..

[34] Bohrey S., Chourasiya V., Pandey A. (2016). Polymeric nanoparticles containing diazepam: Preparation, optimization, characterization, in-vitro drug release and release kinetic study. Nano Converg..

[35] Jarvis M., Krishnan V., Mitragotri S. (2019). Nanocrystals: A perspective on translational research and clinical studies. Bioeng. Transl. Med..

[36] Anselmo A.C., Mitragotri S. (2014). An overview of clinical and commercial impact of drug delivery systems. J. Control. Release.

[37] Dorati R., Detrizio A., Spalla M., Migliavacca R., Pagani L., Pisani S., Chiesa E., Conti B., Modena T., Genta I. (2018). Gentamicin sulfate PEG-PLGA/PLGA-H nanoparticles: Screening design and antimicrobial effect evaluation toward clinic bacterial isolates. Nanomaterials.

[38] Martín-Sabroso C., Fraguas-Sánchez A.I., Aparicio-Blanco J., Cano-Abad M.F., Torres-Suárez A.I. (2018). Critical attributes of formulation and of elaboration process of PLGA-protein microparticles. Int. J. Pharm..

[39] Zhang C., Wu L., Tao A., Bera H., Yang M. (2020). Formulation and in vitro characterization of long-acting PLGA injectable microspheres encapsulating a peptide analog of LHRH. J. Mater. Sci. Technol..

[40] Park K., Otte A., Sharifi F., Garner J., Skidmore S., Park H., Jhon Y.K., Qin B., Wang Y. (2021). Formulation composition, manufacturing process, and characterization of poly(lactide-co-glycolide) microparticles. J. Control. Release.

[41] Doty A.C., Weinstein D.G., Hirota K., Olsen K.F., Schwendeman S.P. (2017). Mechanisms of in vivo release of triamcinolone acetonide from PLGA microspheres. J. Control. Release.

[42] Klose D., Siepmann F., Elkharraz K., Siepmann J. (2008). PLGA-based drug delivery systems: Importance of the type of drug and device geometry. Int. J. Pharm..

[43] Fredenberg S., Wahlgren M., Reslow M., Axelsson A. (2011). The mechanisms of drug release in poly (lactic-co-glycolic acid)-based drug delivery systems—A review. Int. J. Pharm..

[44] Groo A.-C., Matougui N., Umerska A., Saulnier P. (2018). Reverse micelle-lipid nanocapsules: A novel strategy for drug delivery of the plectasin derivate AP138 antimicrobial peptide. Int. J. Nanomed..

[45] Boge L., Umerska A., Matougui N., Bysell H., Andersson M. (2017). Cubosomes post-loaded with antimicrobial peptides: Characterization, bactericidal effect and proteolytic stability. Int. J. Pharm..

[46] Wang X., Wang D., Lu H., Wang X., Wang X., Su J., Xia G. (2024). Strategies to Promote the journey of nanoparticles against biofilm-associated infections. Small.

[47] Modified Surface of PLGA Nanoparticles in Smart Hydrogel, US National Library of Medicine.ClinicalTrials.gov NCT05442736. NCT05442736.

[48] Hen M., Wei J., Xie S., Tao X., Zhang Z., Rana P., Li X. (2019). Bacterial biofilm destruction by size/surface charge-adaptive micelles. Nanoscale.

[49] Zhang Y., Teng D., Mao R., Wang X., Xi D., Hu X., Wang J. (2014). High expression of a plectasin-derived peptide NZ2114 in Pichia pastoris and its pharmacodynamics, postantibiotic and synergy against *Staphylococcus aureus*. Appl. Microbiol. Biotechnol..

[50] Sharifi F., Otte A., Yoon G., Park K. (2020). Continuous in-line homogenization process for scale-up production of naltrexone-loaded PLGA microparticles. J. Control. Release.

[51] Umerska A., Matougui N., Groo A.-C., Saulnier P. (2016). Understanding the adsorption of salmon calcitonin, antimicrobial peptide AP114 and polymyxin B onto lipid nanocapsules. Int. J. Pharm..

[52] Zheng X., Yang N., Mao R., Hao Y., Teng D., Wang J. (2022). Pharmacokinetics and pharmacodynamics of fungal defensin NZX against *Staphylococcus aureus*-induced mouse peritonitis model. Front. Microbiol..

[53] Cruz J., Flórez J., Torres R., Urquiza M., Gutiérrez J.A., Guzmán F., Ortiz C.C. (2017). Antimicrobial activity of a new synthetic peptide loaded in polylactic acid or poly(lactic-co-glycolic) acid nanoparticles against *Pseudomonas aeruginosa*, *Escherichia coli* O157:H7 and methicillin resistant *Staphylococcus aureus*(MRSA). Nanotechnology.

[54] Fan Y., Li X.D., He P.P., Hu X.X., Zhang K., Fan J.Q., Yang P.P., Zheng H.Y., Tian W., Chen Z.M. (2020). A biomimetic peptide recognizes and traps bacteria in vivo as human defensin-6. Sci. Adv..

[55] Wang Z., Liu X., Da T., Mao R., Hao Y., Yang N., Wang X., Li Z., Wang X., Wang J. (2020). Development of chimeric peptides to facilitate the neutralisation of lipopolysaccharides during bactericidal targeting of multidrug-resistant *Escherichia coli*. Commun. Biol..

[56] Yang N., Teng D., Mao R., Hao Y., Wang X., Wang Z., Wang X., Wang J. (2019). A recombinant fungal defensin-like peptide-P2 combats multidrug-resistant *Staphylococcus aureus* and biofilms. Appl. Microbiol. Biotechnol..

